# Leptospirosis is an emerging infectious disease of pig-hunting dogs and humans in North Queensland

**DOI:** 10.1371/journal.pntd.0010100

**Published:** 2022-01-18

**Authors:** Bronwyn Orr, Mark E. Westman, Richard Malik, Auriol Purdie, Scott B. Craig, Jacqueline M. Norris

**Affiliations:** 1 Sydney School of Veterinary Science, The University of Sydney, Sydney, Australia; 2 Elizabeth Macarthur Agricultural Institute (EMAI), Woodbridge Road, Menangle, Australia; 3 Centre for Veterinary Education, The University of Sydney, Sydney, Australia; 4 School of Veterinary and Animal Science, Charles Sturt University, Wagga Wagga, Australia; 5 WHO Leptospirosis Laboratory, Public and Environmental Health, Department of Health, Coopers Plains, Australia; 6 The Sydney Institute for Infectious Diseases, The University of Sydney, Sydney, Australia; UAMS, UNITED STATES

## Abstract

**Background:**

Leptospirosis is a zoonotic disease with a worldwide distribution, caused by pathogenic serovars in the genus *Leptospira*. Feral pigs are known carriers of *Leptospira* species and pig hunting using dogs is a common recreational activity in Queensland, Australia.

**Methodology and principal findings:**

This study aimed to determine the seroprevalence of *Leptospira* spp. serovars in pig-hunting dogs above the Tropic of Capricorn in Queensland and by establishing the geographic distribution, serovars and incidence of human cases of leptospirosis in Queensland, identify potential overlap between human and canine exposure. We also explored the knowledge and risk-taking behaviours of pig-hunting dog owners towards zoonotic diseases.

Ninety-eight pig-hunting dogs deemed healthy by physical examination and owned by 41 people from Queensland had serum submitted for Microscopic Agglutination Testing (MAT) to determine antibody titres against *Leptospira* serovars, while 40/41 dog owners completed a survey on their knowledge of diseases relating to pig hunting. Human leptospirosis cases (n = 330) notified to Queensland Health between 2015–2018 were analysed.

Approximately one quarter (23/87; 26%) of unvaccinated pig-hunting dogs were seropositive to *Leptospira* spp. Although harder to interpret, 8/11 (73%) vaccinated dogs were seropositive to *Leptospira* spp. Pig hunters may be more likely to contract leptospirosis compared with the general Queensland population, based on responses from surveyed hunters. The highest concentration of human leptospirosis was in the wet tropics region of Far North Queensland. There was little overlap between the serovars dogs were exposed to and those infecting humans. The dominant serovar identified in unvaccinated dogs was Australis (13/23; 57%), with serovar Arborea (36/330; 10.9%) responsible for the highest number of human leptospirosis cases. Topaz was the second most common serovar in both humans and dogs and was previously unrecorded in Australian dogs. Most hunters surveyed used hand washing as a zoonotic disease risk reduction technique.

**Conclusions:**

Leptospirosis is an emerging disease of growing significance. The infection requires a ‘one health’ approach to understand its epidemiology. With shifting climatic patterns influencing human-animal-environment interactions, ongoing monitoring of diseases like leptospirosis is critical to helping prevent infection of individuals and disease outbreaks.

## Author summary

Leptospirosis is a disease caused by pathogenic *Leptospira* serovars. *Leptospira* can infect both humans and animals depending on the species and serovar, with disease resulting from direct or indirect exposure to infected urine. In Australia, the highest number of human cases recorded annually occur in the state of Queensland. Feral pig hunting is a popular recreational activity in this state and hunters often use dogs to ‘flush out’ pigs. This study investigated whether dogs used to hunt feral pigs were exposed to *Leptospira* serovars, the geographic distribution of human leptospirosis cases and canine exposure to *Leptospira* serovars in Queensland, and the knowledge of this disease amongst owners of pig-hunting dogs. We found that roughly one quarter of unvaccinated pig-hunting dogs had been exposed to *Leptospira* spp. The wet tropics region of Far North Queensland was found to be the location of highest risk for exposure to *Leptospira* for humans. There was little overlap between the serovars dogs had been exposed to and those reported to have caused disease in humans, except for the serovars Topaz, Australis, Grippotyphosa, and Pomona. Finally, pig hunters were found to be at increased risk of being diagnosed with leptospirosis compared to the Queensland public and engaged in risky hunting practices such as not wearing personal protective equipment (PPE). Our findings reinforce the emerging significance of leptospirosis for physicians and veterinarians in Australia.

## Introduction

Leptospirosis is a zoonotic disease with a worldwide distribution and emerging significance [1, 2]. It is caused by pathogenic serovars in the bacterial genus *Leptospira*, including some capable of infecting both humans and animals [3]. These spirochetes have the potential to infect the kidneys of mammals after direct exposure to urine from an infected mammal, or indirectly via soil and water contaminated with infected urine [4]. Critically, leptospirosis can have widely different clinical manifestations depending on the size of the inoculum, infecting serovar, host species and pre-existing host immunity [5]. Mammalian species can be either incidental hosts, which may or may not show clinical signs of disease, or maintenance hosts, which are usually infected without showing clinical signs, but contribute significantly to organism shedding and transmission [6].

The genus *Leptospira* has a long history of changing taxonomic and genomic classification. Currently the genus is considered to have two clades (pathogenic and saprophytic), with each clade further divided into subclades (P1, P2, S1 and S2) [7]. Subclade P1 contains 64 pathogenic species including those commonly infecting humans and animals [7]. *Leptospira* spp. can also be classified into serovars, which have limited taxonomic utility but can play an important role in epidemiologic investigations. Each *Leptospira* serovar has a distinct bioclimatic niche, with defined predilections for incidental and maintenance hosts [5]. It is this diversity, as well as the dose of the infective inoculum and immune competence and species of the host, that contribute to the variable clinical presentations encountered in infected human and veterinary patients [8]. For this reason, research conducted in different bioclimatic regions and countries may not be comparable if local serovars and maintenance host populations differ substantially [9].

Across Australia, cattle, pigs, horses, dogs, cats, macropods, possums, bandicoots, rats, mice, seals and Tasmanian devils have been found to carry *Leptospira* spp. [10–15]. With many unique native animal species, it is crucial to determine the epidemiology of leptospirosis within an Australian context. The pattern of disease seen in association with *Leptospira* serovars is likely to be influenced by a changing climate, thus determination of the current geographic distribution of leptospirosis assists with disease preparedness in a changing world.

Queensland (Qld) is the second largest state in Australia by land area. At more than 1.7 million square kilometres, it is roughly 4.8 times bigger than Germany and annually records the highest number of human leptospirosis cases in the country. Although leptospirosis is considered an occupational risk for farmers, veterinarians, meat workers and military personnel, it is increasingly recognised as a risk for campers, white water rafters and those who participate in sport or other outdoor activities in potentially contaminated areas [16]. It is a notifiable disease for human patients in Qld, but not for companion or production animal species [16, 17].

All probable and confirmed human cases of leptospirosis are reported to Queensland Health’s Public Health Units in accordance with the *Public Health Regulation 2018*. The World Health Organisation (WHO)/Office International des Epizooties (OIE) Leptospirosis Reference Laboratory for Australia is based in Brisbane, Queensland’s capital [18]. This reference laboratory, which serotypes most notified cases of leptospirosis in Australia, tests serum against a panel of 22 serovars, including 16 local and six transboundary serovars, using Microscopic Agglutination Testing (MAT). The passive nature of leptospirosis surveillance means little is known about the geographic distribution of serovars within Australia.

Feral pigs (*Sus scrofa*) are an important pest species in Queensland, where a large proportion of Australia’s estimated 3.5–24 million feral pigs are located [19, 20]. Feral pigs were introduced by European colonists in the early 1800s and cause extensive damage to crops and native forests [19]. As a declared pest, Australian landowners have a legal responsibility to remove pest animals such as pigs from their land by poisoning, shooting or other means. Partly as a result of this, pig hunting has become a popular activity in Queensland, particularly in Central, North and far North Queensland, with many hunters using dogs to flush pigs out of difficult to access land [21]. Feral pig hunting is a known risk factor for humans contracting brucellosis caused by the zoonotic bacterium *Brucella suis* [22, 23]. Dogs (*Canis lupus familiaris*) used for pig hunting have also been shown to contract *B*. *suis* during hunts or when fed the uncooked flesh of feral pigs [24].

Leptospirosis is an emerging infectious disease in Australian dogs. Historically, it has been understudied. Following outbreaks between 2018 and 2021 in Sydney and Melbourne, Australia’s two largest cities, with a mortality rate in dogs of 88%, canine leptospirosis has captured the attention of Australia’s veterinary and dog-owning communities [25].

There are two canine vaccines currently available in Australia to help prevent development of disease due to *Leptospira* infection. An Australian Pesticides and Veterinary Medicines Authority (APVMA) registered, killed, adjuvanted vaccine produced by Boehringer Ingelheim (Protech® C2i) [26] is used to protect against disease caused by serovar Copenhageni for up to 12 months. An APVMA restricted use, killed, adjuvanted bespoke vaccine (AUSLEPTO, Tréidila Biovet) [27] can be used on dogs in Queensland and the Northern Territory to help protect against disease caused by serovar Australis for up to 12 months. Both vaccines can be given concurrently in high-risk areas like Queensland, although uptake is currently unknown and no cross-protection between serovars is thought to be provided. While vaccination against *Leptospira* is not considered ‘core’ by the Australian Veterinary Association or World Small Animal Veterinary Association [28, 29], the recommendation has always been to ‘*use in geographical areas where a risk of exposure has been established or for dogs whose lifestyle places them at risk*’ [29]. Until recently, vaccination against leptospirosis was not recommended outside of North Queensland and the Northern Territory [30].

As feral pigs are known carriers of *Leptospira* spp., particularly *Leptospira interrogans* serovar Pomona [31], we hypothesised that dogs used for pig hunting would be exposed during hunts to this and other *Leptospira* spp. shed in the environment by other animals. Pig-hunting dogs have been shown to act as sentinels for other diseases affecting the wider canine population [32], therefore we aimed to determine if they could also act as a sentinel population for leptospirosis in both canine and human populations.

This study therefore aimed to determine the seroprevalence of *Leptospira* spp. serovars in pig-hunting dogs above the Tropic of Capricorn in Queensland and by establishing the geographic distribution, serovars and incidence of human cases of leptospirosis in Queensland, identify potential overlap between human and canine exposure. People travel regularly for work and recreation, such that some human cases of leptospirosis are acquired in locations outside their local region. The serovars detected in human cases of leptospirosis may shed light on the serovars that pig-hunting dogs are exposed to above the Tropic of Capricorn. Furthermore, although seropositivity suggests past exposure, it can be used to evaluate the burden of disease in an environment. Comparing human leptospirosis with canine *Leptospira* spp. exposure can therefore reveal potential ‘hot spots’ or regions of increased risk. We also explored the knowledge and risk-taking behaviours of pig-hunting dog owners towards zoonotic diseases to determine whether pig-hunters may be at an increased risk for developing clinical or subclinical leptospirosis.

We explored whether pig-hunting dogs in Queensland have high rates of *Leptospira* spp. exposure; whether common serovar exposures would be evident in humans and canines from the same geographic areas in Queensland; and whether less than 50% of pig-hunting dog owners would take any steps to reduce their risk of exposure to zoonotic diseases. These enquiries were based on personal experience and previous research into pig-hunting dogs [21, 32].

## Materials and methods

### Ethics statement

Ethics approval for obtaining notified human cases of leptospirosis was given by The University of Sydney Human Research Ethics Committee (approval number 2019/394). Ethics approval for the survey of hunters was obtained from The University of Sydney Human Research Ethics Committee (approval number 2018/317). Ethics approval for all animal handling and sampling was obtained from The University of Sydney Animal Ethics Committee (approval number 2018/1341).

### Sample population—dogs

**Fig 1:**
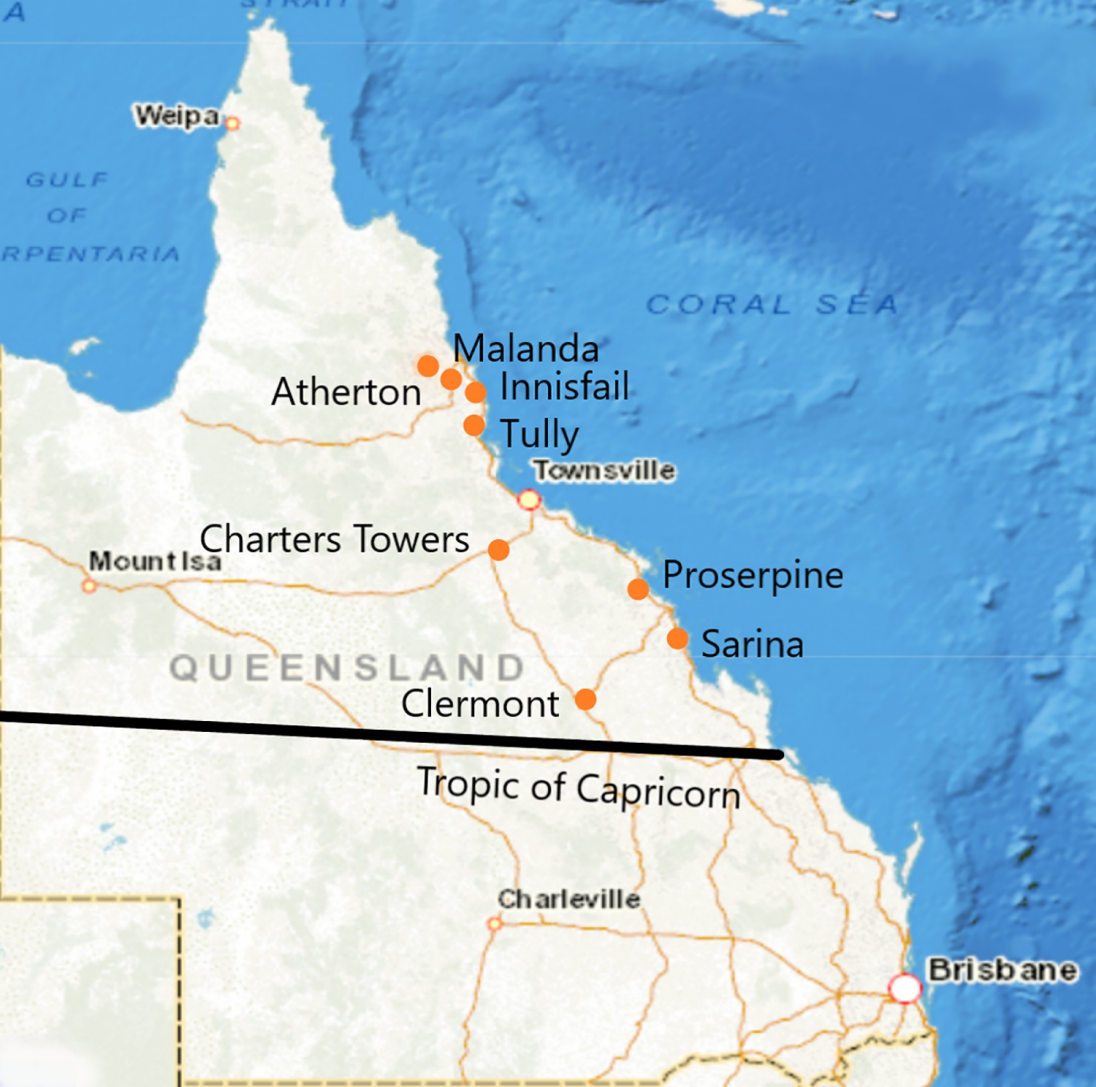
**Location of the eight participating veterinary clinics in a serosurvey of pig-hunting dogs from above the Tropic of Capricorn (black line) in the state of Queensland, Australia** (Source: https://d-maps.com/carte.php?num_car=63942&lang=en).

Ten veterinary clinics above the Tropic of Capricorn in Central, North and Far North Queensland were approached first by email, then in person, to participate in this study. Clinics were chosen based on location, as clinics in rural and regional areas were more likely to have pig hunter clientele. Eight clinics participated in the study, enrolling dogs from the regions around Sarina, Clermont, Proserpine, Charters Towers, Tully, Innisfail, Malanda, and the Atherton Tableland (Fig 1). All dogs enrolled were older than six months-of-age. The key inclusion criteria specified that the dog must have been currently used for pig hunting. Participating veterinary clinics provided medical histories for each patient, and the following data were captured: the dog’s age, breed, sex, reproductive state (sexually intact *vs* neutered) and vaccination history at the time of sampling. Dogs were considered vaccinated for *Leptospira interrogans* serovar Copenhageni and/or *Leptospira interrogans* serovar Australis if they had received appropriate vaccination in accordance with WSAVA vaccination guidelines [29], within 12 months of sampling, which took place over a four-month period between August and November 2018.

A registered veterinarian performed a physical examination and declared each dog to be healthy prior to sampling. Free-catch urine samples were collected where possible, then frozen and stored at -20°C before being sent to a commercial laboratory (IDEXX Laboratories) for real-time polymerase chain reaction (qPCR) testing for *Leptospira* spp. DNA. Whole blood (3–5 mL) was collected via cephalic venepuncture, stored on ice, and allowed to clot; serum was harvested, aliquoted and stored at -20°C prior to submission to the Australian reference laboratory for MAT (S1 Table). An agglutination result at 1/50 dilution or higher was considered a potentially positive titre in accordance with the reference laboratory standards [15, 33]. Dogs that recorded multiple positive titres were assigned primary serovar(s) based on the highest titre(s) and absence of known cross reactions to the serovar with the highest titre. Known cross reactions include Australis with Copenhageni; Copenhageni with Zanoni; Zanoni with Robinsoni; Pomona with Djasiman and Cynopteri; and Grippotyphosa with Australis. Serovars without known cross reactions were recorded as independent positive results. As vaccination is suspected of causing antibodies detectable on MAT [34–36], canine results were divided into vaccinated and unvaccinated groups.

### Sample population—dog owners

Owners self-identified their dogs as being used for pig hunting. They completed a survey while a blood sample was obtained from their dog. The survey (S1 Text) was paper-based and comprised 34 questions: four open questions and 30 closed or semi-closed questions. The survey was broadly divided into five themes: demographic information about the respondent; information about their hunting dogs; hunting style and geographical location of their hunts; general health of their dogs; and knowledge and awareness of zoonotic disease risks including use of personal protective equipment (PPE) during hunts.

### Sample population–human cases of leptospirosis

Data on leptospirosis in human patients were obtained from the Queensland Health Epidemiology Unit via a Public Health Act request for notified cases of leptospirosis between January 2015 and December 2018. These dates captured sufficient human cases for robust analysis while encompassing the sampling and exposure period of the canine study participants. The national case definition for leptospirosis in Australia, as defined by the Commonwealth Department of Health [37], is that only confirmed cases should be notified. Confirmed cases of leptospirosis require definitive laboratory evidence of infection. Definitive evidence falls into one of three areas:

1. Isolation of pathogenic *Leptospira* species in cell culture, or
2. A four-fold or greater rise in *Leptospira* MAT between acute and convalescent phase sera obtained at least two weeks apart and preferably conducted at the same laboratory, or
3. A single *Leptospira* MAT ≥ 400 supported by a positive enzyme-linked immunosorbent assay (ELISA) IgM result.

In Queensland, however, all suspected or laboratory confirmed cases of leptospirosis are reported to the epidemiology unit. Queensland Health also includes laboratory evidence, such as detection of *Leptospira* by nucleic acid testing, in their notification criteria.

De-identified data obtained from Queensland Health on notified cases of leptospirosis included age, gender, Statistical Area 2 region (SA2; medium-sized areas that represent a community which interacts together socially and economically; the smallest area able to be released without risking patient identification), date the disease was notified to authorities and date of disease onset. In some cases, the serovar was also identified.

### Data analysis

Data was mapped using National Map (https://nationalmap.gov.au/), an Australian Government open-source software for mapping using Statistical Area Levels. The age and seroprevalence in unvaccinated and vaccinated dogs were compared using Mann-Whitney tests. Fisher’s exact tests were used to compare the likelihood that vaccinated and unvaccinated dogs registered a positive *Leptospira* spp. titre and was also used to compare the rate of leptospirosis in surveyed pig hunters with the Queensland population. A significance level of P < 0.05 was used for all statistical tests.

## Results

### Sample population–dogs

Serum samples were obtained from 98 pig-hunting dogs, owned by 41 different people. Urine samples were collected from 21 of the 98 pig-hunting dogs. Completed paper surveys were received from 40/41 pig-hunting dog owners. The medical records of all 98 dogs were retrieved from the recruiting veterinary clinics.

Ten urine samples from *Leptospira* seropositive dogs were tested for *Leptospira* spp. DNA and all returned a negative result.

A total of 31 dogs (31/98; 32%) recorded *Leptospira* spp. antibodies to at least one serovar at a titre of 1/50 or greater. As some dogs were vaccinated against serovars Australis and/or Copenhageni, these results were therefore further divided into unvaccinated dogs and vaccinated dogs.

#### i) Canine seroprevalence (unvaccinated dogs; n = 87)

**Fig 2:**
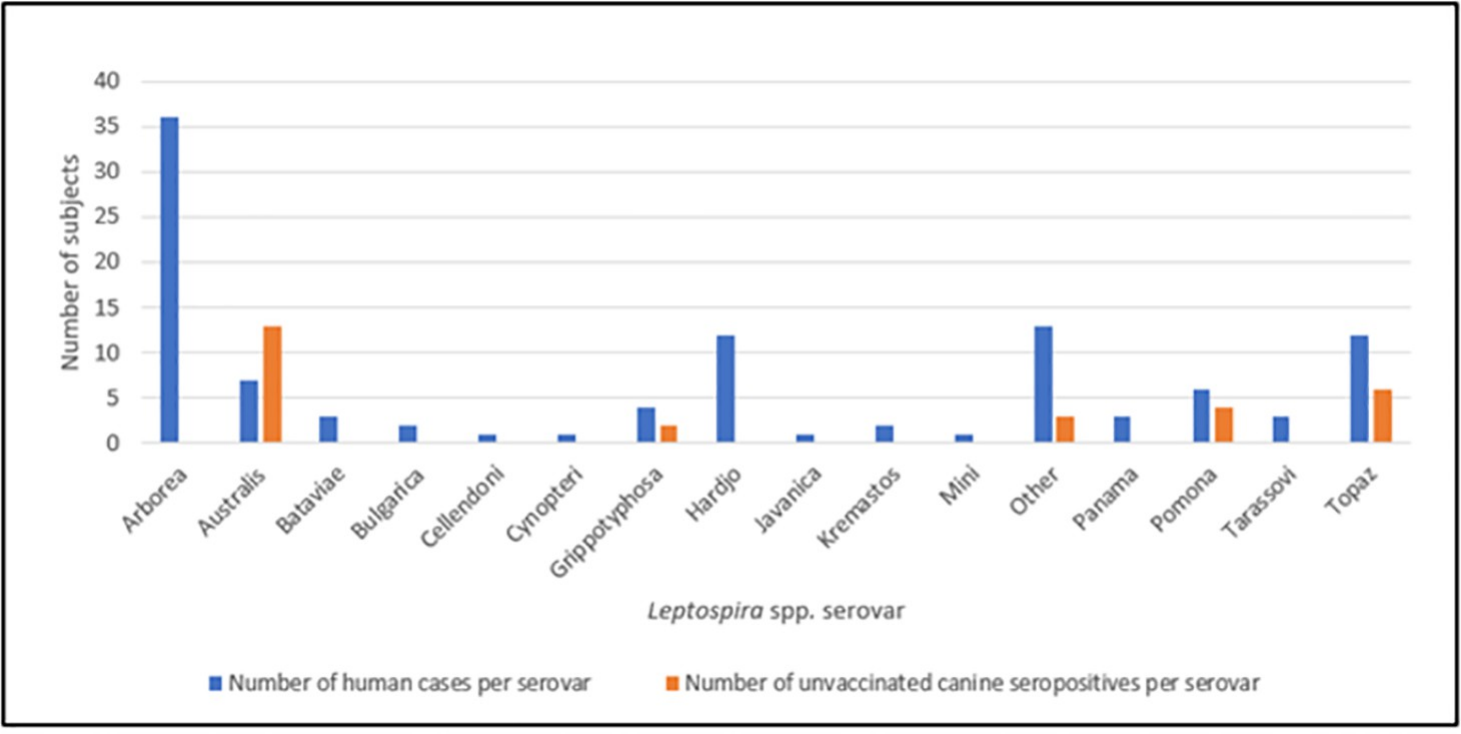
Serovars of leptospirosis cases in humans notified to Queensland Health from 2015–2018 (blue histograms) and primary serovar(s) of 23 unvaccinated pig-hunting dogs sampled for *Leptospira* spp. (orange histograms) as part of a serosurvey in 2018 from above the Tropic of Capricorn in Queensland, Australia. Five dogs had more than one primary *Leptospira* serovar detected and ‘other’ includes serovars such as Zanoni, Szwajizak, Djasiman and Canicola.

Of 87 unvaccinated pig-hunting dogs, 23 (26%) recorded a positive *Leptospira* titre on MAT, with seven dogs (8%) recording a titre of 1/400 or greater (Table 1). Seropositive unvaccinated dogs (median age 3 years; IQR 2.8–5.5 years) were significantly older than seronegative unvaccinated dogs (median age 2 years; IQR 1.88–3.5 years) (P = 0.032; Mann-Whitney *U-*test). Neutering rates between seronegative (7/64; 11%) and seropositive (3/23; 13%) unvaccinated dogs were not significantly different (P = 0.72). Fig 2 shows the diversity of primary serovars detected in the unvaccinated canine cohort.

**Table 1:** Signalment, *Leptospira* serovar and titre of unvaccinated pig-hunting dogs with positive *Leptospira* titres.

Breed or type	Age (years)	Sex	*Leptospira* serovars detected*	Titre of primary serovar(s)
Pitbull X Boxer	3	FN	**Australis**	1/50
Bull Arab X	3	M	**Pomona**; Cynopteri	1/50
Bull Arab X Cattle Dog	6	F	**Szwajizak**	1/50
Bull Arab X	7	F	**Topaz**	1/50
Bull Mastiff X Bull Arab	3	M	**Pomona; Grippotyphosa;** Cynopteri; Djasiman	1/100; 1/50
Mastiff X	7	M	**Pomona**; Cynopteri; Djasiman	1/100
Bull Arab	13	M	**Zanoni**	1/100
Ridgeback X	0.5	M	**Grippotyphosa**	1/100
Bull Terrier X Bull Arab	3	F	**Australis**	1/100
Smithfield Cattle Dog X	3	M	**Australis; Topaz**	1/200; 1/100
Bull Arab X Staffy	6.5	M	**Australis; Topaz**	1/200; 1/100
Great Dane X Mastiff	3	M	**Australis**	1/200
Bull Mastiff X	9	M	**Australis**	1/200
Cattle Dog X	4	F	**Szwajizak**	1/200
Mastiff X Great Dane	5	M	**Topaz**	1/200
Bull Terrier X Cattle Dog	3.5	F	**Australis; Topaz**	1/200; 1/200
Bull Arab	2.5	F	**Pomona**; Cynopteri; **Topaz**; Copenhageni; Zanoni; Djasiman; Bulgarica; Grippotyphosa	1/400; 1/200
Ridgeback X	6	FN	**Australis;** Grippotyphosa	1/400
Cattle Dog X	2	F	**Australis**	1/400
Cattle Dog X	2	M	**Australis**; Canicola; Szwajizak; Cynopteri; Panama	1/800
Bull Arab X Catahoula	3	M	**Australis**	1/800
Greyhound X Staghound	3	MN	**Australis**	1/800
Bull Arab	1	M	**Australis**	1/1600

* (titre >1/50) with primary serovar(s) in bold; abbreviations: X crossbred; M male intact; F female intact; FN spayed female; MN male castrated

#### ii) Canine seroprevalence (vaccinated dogs; n = 11)

Eight of 11 (73%) vaccinated dogs recorded a *Leptospira* titre above 1/50. Ten dogs were vaccinated against both Copenhageni and Australis, while one dog was vaccinated against serovar Australis only. Compared with unvaccinated dogs, vaccinated dogs were significantly more likely to register a positive *Leptospira* titre (23/87 vs 8/11; P = 0.038; two-tailed Fisher’s Exact test). Only two primary serovars were reported in the 11 vaccinated dogs, Australis and Zanoni, with one dog recording a 1/1600 titre to serovar Zanoni (S2 Table). Vaccinated dogs were significantly older (median age 6 years; IQR 5–8 years) than unvaccinated dogs (median age 3 years; IQR 2–4 years) (P = 0.001; Mann-Whitney *U-*test). Three vaccinated dogs (27%) had no detectable circulating anti-*Leptospira* spp. antibodies.

#### Sample population—dog owners

Table 2 demonstrates the awareness pig hunters reported of the risks of zoonotic disease as a result of pig hunting. Further information on the questions posed to pig hunters with dogs is found in S1 Text. Two of the 40 (5%) dog owners stated they had previously had leptospirosis, which is significantly higher (P < 0.00001; Fisher’s exact test) than the notification rate of leptospirosis in Queensland (1.85 cases per 100,000 people) [38]. Table 3 demonstrates that 70% of hunters (28/40) reported using hand washing as a risk-reduction strategy, although only 38% (15/40) reported wearing protective clothing and only one (1/40; 3%) reported wearing gloves during hunts.

**Table 2:** Survey of 40 pig-hunting dog owners in 2018 regarding zoonotic disease risk posed by pig hunting with dogs.

Question	Answer (number of respondents)
Q30 Have you ever become sick and been diagnosed with Q fever, brucellosis or leptospirosis?	Yes (2)
	No (36)
	Not sure (2)
Q31 If yes, which disease(s) have you been diagnosed with?	Leptospirosis (2)
Q32 Did you know that feral pigs can carry all three of these diseases?	Yes (35)
	No (5)
Q33 Did you know that pig-hunting dogs can become infected with all three of these diseases?	Yes (34)
	No (6)

**Table 3:** Results from the survey of 40 pig-hunting dog owners in 2018 to Question 34, ‘Do you take any precautions when killing pigs to avoid contracting these diseases [Brucellosis, Q Fever, Leptospirosis]?’.

Precaution	Number (percentage) of hunters that take precautions during hunts
None	6 (15%)
Wash hands	28 (70%)
Wear protective clothing (e.g., long shirts)	15 (38%)
Wash equipment	8 (20%)
Avoid feral pig organs	13 (33%)
Wear gloves	1 (3%)

### Sample population–Human cases of leptospirosis

A total of 330 human cases of leptospirosis were reported during the period January 2015 to December 2018, with 208 cases occurring in the study area of Central and North Queensland, as per SA2 regional definitions (SA2 regions above the Tropic of Capricorn). Approximately one third of human cases (107/330; 32%) had serovars recorded (Fig 2). Serovar Arborea was responsible for more than one third of human cases (36/107; 34%), with serovars Hardjo and Topaz the next most common infecting serovars (both 12/107; 11% each).

#### Geographic distribution

**Fig 3:**
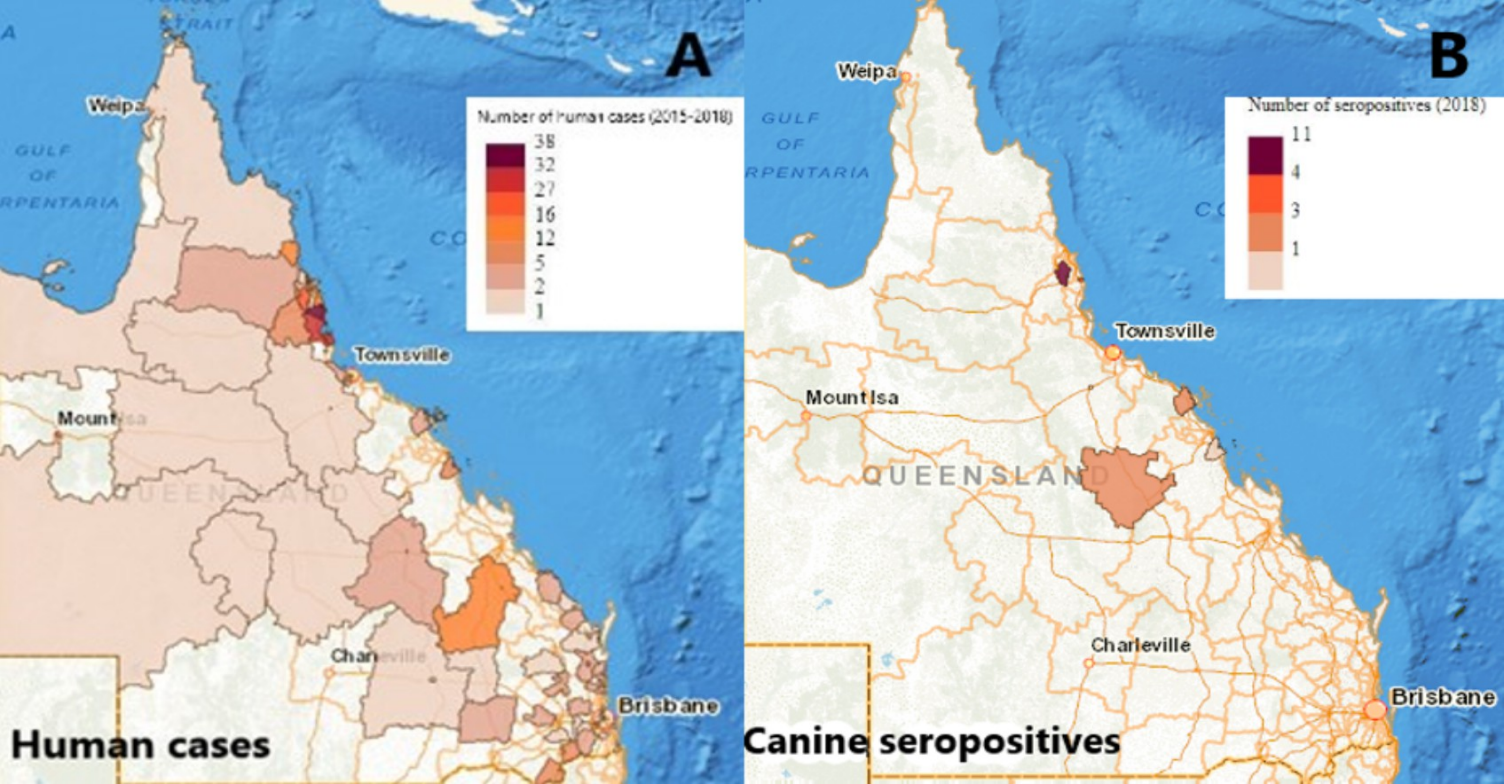
**(A) Distribution of human cases of leptospirosis notified to Queensland Health from 2015–2018; (B) Distribution of unvaccinated canine seropositives on microscopic agglutination testing (MAT) for *Leptospira* species from a 2018 serosurvey of pig-hunting dogs** (Source: https://www.nationalmap.gov.au/. Commonwealth of Australia (Geoscience Australia) 2020. Released under Creative Commons Attribution 4.0 International License).

Fig 3 shows the concentration of seropositive dogs and human leptospirosis cases in the wet tropics region of Far North Queensland. The five SA2 regions with the highest number of human leptospirosis cases were Johnstone (38 cases), Tully (32 cases), Innisfail (27 cases), Malanda-Yungaburra (16 cases) and Daintree (12 cases). These regions cover an area of approximately 3000km^2^ in the wet tropics region of Queensland.

**Fig 4:**
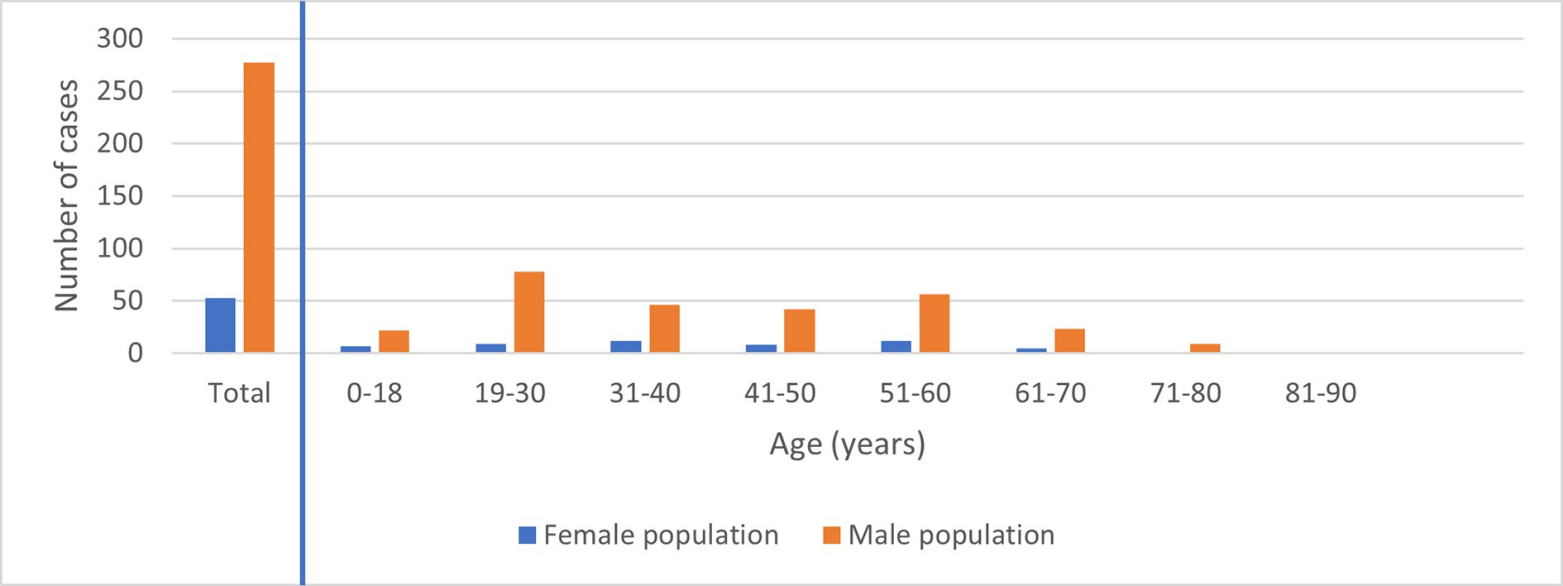
Gender distribution of human leptospirosis cases notified to Queensland Health from 2015–2018.

The distribution of human leptospirosis cases across different age groups in males and females is illustrated in Fig 4. Of the 330 leptospirosis cases in Queensland, 84% were male (277/330), with males between 19–30 years old accountable for almost a quarter of the cases (78/330; 24%).

## Discussion

Leptospirosis is an important but incompletely understood disease of domestic animals and humans above the Tropic of Capricorn in Queensland, Australia. The interactions between host species, maintenance species and the environment [38], combined with numerous serovars and probable unknown wildlife reservoir species, adds complexity. This makes increasing our knowledge of the epidemiology of leptospirosis critical to understanding the risks faced by people and dogs that live in the tropical north, particularly as human cases appear to be increasing [39].

Determining the seroprevalence of *Leptospira* spp. serovars in pig-hunting dogs above the Tropic of Capricorn in Queensland helps our understanding of the role their lifestyle and exposure to both the environment and feral pigs has on seropositivity. Due to the uncertain impact of the two available canine vaccinations in Australia on *Leptospira* spp. seropositivity and MAT cross-reactivity, results from the serosurvey were split and analysed based on vaccination status. Unvaccinated dogs, which formed the majority of the sampled population, present an opportunity to analyse canine MAT results free of the confounding effects of vaccination.

Currently, Australia has two monovalent vaccines available commercially for dogs for protection against *Leptospira* serovars Copenhageni and Australis, with the Australis vaccine not used outside Australia. Both vaccines are thought to reduce the severity of disease rather than actually preventing infection [32, 33]. Neither vaccine has supporting data available from the respective manufacturers concerning the antibody and cell-mediated immune response to vaccination by dogs in the field, and this is a research gap that requires attention.

Of the 23 unvaccinated pig-hunting dogs seropositive to *Leptospira* spp. (Table 1), seven had *Leptospira* titres above 1/400, with three recording titres of 1/800 and one recording a titre above 1/1600. All the dogs in this study were considered clinically normal at the time of venepuncture based on an unremarkable physical examination. There is established doctrine in human leptospirosis diagnostic criteria that one titre of >1/400 for a pathogenic serovar is considered diagnostically positive for leptospirosis [16, 37]. However, this doctrine hasn’t been established in dogs. We believe a >1/400 titre may be considered a presumptive positive in unvaccinated dogs, potentially reflecting recent clinical or subclinical disease or current subclinical (asymptomatic) disease, or both [40]. However, the absence of *Leptospira* spp. DNA in the 10 urine samples from a range of seropositive dogs (including four with titres in excess of 1/400) suggests a genuine absence of active clinical disease at the time of testing. It should be noted that urinary shedding can be intermittent, therefore active infection with *Leptospira* spp. could not be ruled out as a reason for the observed high *Leptospira* titres [41]. While MAT is the preferred serological test in dogs, more research should be conducted on establishing the cut-off titre for a presumptive positive diagnosis in this species. Unvaccinated seropositive dogs were significantly older than unvaccinated seronegative dogs, consistent with a cumulative lifetime exposure risk to *Leptospira* spp. for hunting dogs [42].

The unvaccinated pig-hunting dogs sampled registered high titres (>1/400) against serovars Australis, Topaz, Pomona, Szwajizak, Grippotyphosa and Zanoni (Fig 2). Excluding serovar Pomona, which is known to be carried by feral pigs, it appears the environment above the Tropic of Capricorn represents a key *Leptospira* spp. exposure risk for pig-hunting dogs as the other serovars are carried by native marsupials, rats, mice and cattle [33, 43–47]. With some overlap between human and canine serovars, particularly serovars Topaz, Australis, and Pomona, it is possible pig-hunting dogs are acting as sentinels, or even incidental hosts, for human leptospirosis. The Centre for Disease Control in the USA recognises pet dogs as a possible source of *Leptospira* spp. exposure in humans [48], although dogs are generally considered to be an uncommon source of infection in humans [6, 49, 50].

Topaz has, prior to the present study, been unrecorded in dogs in Australia, although it has been found in cattle, bandicoots, and kangaroos [44, 51]. Our data found it was the second most common primary serovar in our canine population as well as a corresponding human population. It is unknown what role serovar Topaz plays in canine clinical disease, although it’s relatively recent discovery in 1990 and it’s increasing prevalence in human notifiable cases [51], might suggest the environmental exposure risk for Topaz infection is increasing. It would also be worthwhile conducting further research into the role of this serovar in clinical disease in dogs.

The most common serovar recorded in humans in Queensland between 2015 and 2018 was Arborea (Fig 2). Serovar Arborea is found in rats and mice across the world, and since its first detection in Queensland in 1998, it has been found throughout the state [52]. It is often detected in patients involved in agricultural industries, as is serovar Hardjo, which was also frequently detected in human patients in Queensland. Serovar Hardjo also has a worldwide distribution and is primarily carried by cattle [47, 53–56]. Interestingly, serovar Australis, the most detected serovar in dogs, was only responsible for seven (6.5%) human cases of leptospirosis. There must be a logical reason for the difference in serovar prevalence in humans compared with dogs, but currently we lack the insight to understand this aspect of the epidemiology. Similarly, we currently do not understand why serovar Arborea is comparatively rare in dogs while being so common in human patients.

It is possible the geographic distribution of serovars plays a role in the serovar prevalence between humans and dogs. North and Far North Queensland are arguably the leptospirosis ‘hot spots’ for Australia in terms of human disease. Although sparsely populated, with a small permanent human population, the region is responsible for a substantial proportion of Australia’s human leptospirosis notifications every year [38]. A quarter (21/82; 25.6%) of all research papers on leptospirosis in humans in Australia concern this geographic region, even though less than 3% of Australia’s population lives there [57]. Notifiable cases of human leptospirosis from 2015–2018 were concentrated in Far North Queensland, with some overlap in canine unvaccinated seropositive cases (Fig 3). North and Far North Queensland includes the UNESCO Wet Tropics World Heritage Area. Tropical towns in this region such as Babinda and Innisfail receive annual rainfall in excess of 3,560 mm [58]. As a result, pooling ground water and run-off is present near continuously for several months of the year. These conditions are ideal for the spread of *Leptospira* spp., which can survive for up to 152 days in fresh water [5]. Additionally, the wet tropics is incredibly biodiverse, which intensifies the complex interactions between humans, animals and the environment [59].

Until now, there have only been 13 published studies on *Leptospira* spp. exposure and disease in dogs in Australia [12, 13, 25, 60–69]. Four studies were serosurveys [12, 60, 61, 64], which provides limited information on historical canine exposure to *Leptospira* spp. serovars. The usefulness of the data from these older studies in comparison with contemporary canine populations is limited. Since the first canine serosurvey was published in 1952, there have been significant changes to serovar typing and serogroup taxonomy, as well as the set points for positive titre cut-offs and serological methodology. This makes comparing serosurveys across time challenging.

Additionally, single timepoint serosurveys demonstrate past and current exposure but do not give information on the timing of exposure or whether the detected serovar caused clinical or subclinical disease at that time [70]. Future studies investigating pig-hunting dogs should include ‘control’ populations of farm or companion dogs to determine the influence hunting has compared with other interactions with the environment, such as herding livestock [71, 72]. Pig-hunting dogs often travel to hunting locations outside of their home base, which makes isolating the exact location of infecting serovars difficult. The high level of seropositivity in this cohort of pig-hunting dogs suggests hunting dogs in Central, North and Far North Queensland may have an increased risk of being exposed to the agents responsible for leptospirosis.

Vaccination should result in antigen-specific antibodies being produced by B lymphocytes and plasma cells, with long-term immunity requiring either persistence of antibody titres above a protective threshold and/or the stimulation and maintenance of immune memory B and T cells [73]. Studies in dogs concerning several polyvalent *Leptospira* spp. serovar vaccines, covering serovars Canicola, Grippotyphosa, Icterohaemorrhagiae and Pomona, have shown high immediate post-vaccinal titres of up to 1/6400 can be achieved [35, 36], followed by a substantial drop six months post-vaccination [36, 65], with some dogs registering a poor anamnestic response by only one month post-vaccination [36]. Of the 11 vaccinated dogs in the current study, 27% (3/11) had no circulating antibodies; but this does not mean memory B and T cells had not been primed, particularly as killed vaccines, like the Australis and Copenhageni vaccines, promote a humoral immunity resulting in decreasing antibody titres over time [34, 74].

The potential cross reaction between Australis and Copenhageni makes interpreting post-vaccination results challenging, as both currently available canine vaccines are directed against one or the other of these serovars. The high proportion of vaccinated dogs seropositive to Zanoni (5/11; 45.5%) compared with unvaccinated dogs (1/87; 1.1%), suggests there may be an unknown cross reaction occurring between either of the canine vaccines and Zanoni on the MAT. Several serovars have known cross-reactions on MAT, and there are likely unknown additional cross reactions in dogs.

Our data showed that human leptospirosis in Queensland is a disease that predominantly affects men (84%, 277/330). This is not unexpected, as the occupations most at risk like farmers, meat workers or the military, are male dominated [38, 75, 76]. The age group at greatest risk of contracting leptospirosis appears to be between 18–30 years of age (Fig 4). Previously research has found that pig hunters in Queensland who use dogs also fit this demographic of young men [21, 77].

The average annual notification rate of leptospirosis in Queensland between 2007 and 2016 was 1.85 cases per 100,000 population, or 0.002% of the population annually [38]. Recreational exposure is an emerging risk [38, 39], and our finding that 5% (2/40) of pig hunters surveyed had apparently contracted leptospirosis (Table 2), indicates that pig hunting may increase the risk of exposure to *Leptospira* spp. It is possible that pig hunting (slaughtering animals, butchering carcases) *per se* and activities commonly associated with pig hunting (camping, wading through wet vegetation such as cane fields) both increase exposure to *Leptospira* spp. [22]. Alternatively, our findings that pig hunters may be more likely to contract leptospirosis compared with the general population, highlights that such individuals could have additional risk factors compared to the general population, such as being employed in farming or meat processing or belonging to the key demographic of young men. Further research should be done to determine if the activity of pig hunting increases the risk of human leptospirosis, as it is currently not identified as a risk factor by Queensland Health [39].

As recreational activities are an emerging risk for leptospirosis in Australia, more research is justified on the impact of leptospirosis on hunters and whether risk-reduction activities can play a role in improving human health outcomes [78]. Our research showed that 15% (6/40) of pig hunters take no simple precautions to reduce the risk of zoonotic disease during hunts, with 70% (28/40) washing their hands during hunts but only 3% (1/40) wearing gloves while hunting (Table 3). However, hunter awareness of the risks posed by zoonotic diseases carried by feral pigs and hunting dogs was high (Table 2). There appears to be an opportunity to utilise a health promotion strategy to bridge the knowledge-action gap among hunters to devise practical risk-reduction strategies that limit pig hunters’ risk of contracting zoonotic diseases including leptospirosis.

As climate change is predicted to increase flooding and monsoonal conditions in Queensland [79], the significance of the environment in leptospirosis transmission is likely to increase [55]. In Australia, known *Leptospira* spp. maintenance hosts like feral pigs, rodents and marsupials interact with wet conditions to create a ‘perfect storm’ for incidental hosts like humans and dogs, who are exposed through recreational activities such as pig hunting and camping. Further research should be conducted to determine exposure risks to *Leptospira* spp. in humans and the likelihood of exposure resulting in disease. The real rate of leptospirosis in Queensland is likely underreported [55, 80], and increased detection and reporting of identified serovars would assist with epidemiologic modelling, particularly in a changing climate.

There may also be significant benefits in making canine leptospirosis a notifiable disease in Queensland, given the important role the state plays in maintaining the disease in Australia and the zoonotic potential. Ensuring all canine cases of leptospirosis were notified to veterinary and public health authorities would provide important data for disease monitoring, as well as providing accurate data on infecting serovars to allow the registration or development of more suitable canine vaccines.

Effective human vaccination against leptospirosis remains an elusive goal, with issues relating to serovar diversity, short duration of immunity and severe side effects needing to be overcome [81]. However, vaccination of dogs against leptospirosis is readily available, and our results show that dogs in North and Far North Queensland should at least be vaccinated against serovar Australis to prevent severe disease. Future research on the role serovar Topaz plays in clinical canine leptospirosis should be explored, as it appears dogs in North Queensland are being increasingly exposed to this serovar.

## Supporting information

S1 Table *Leptospira* species serovars tested in the microagglutination test (MAT) panel (*n* = 22). (DOCX)

S2 Table Signalment and detailed *Leptospira* serological results of 11 pig-hunting dogs vaccinated against *Leptospira*. (DOCX)

S1 Text Survey completed by pig-hunting dog owners. (DOCX)
