# Supplementary material for: Leptospirosis is an emerging infectious disease of pig-hunting dogs and humans in North Queensland
Source: PLoS Negl Trop Dis. 2022 Jan 18;16(1):e0010100. doi: 10.1371/journal.pntd.0010100 (PMC8797170; doi:10.1371/journal.pntd.0010100)
Supplement: S1 Table — (DOCX) [file pntd.0010100.s001.docx]

**S1 Table: *Leptospira* species serovars tested in the microagglutination test (MAT) panel (*n* = 22)**

| Species | Serovar |
| --- | --- |
| *Leptospira borgpetersenii* | Arborea |
| *Leptospira interrogans* | Australis |
| *Leptospira interrogans* | Bataviae |
| *Leptospira kirschneri* | Bulgarica |
| *Leptospira interrogans* | Canicola |
| *Leptospira weilii* | Celledoni |
| *Leptospira interrogans* | Copenhageni |
| *Leptospira kirschneri* | Cynopteri |
| *Leptospira interrogans* | Djasiman |
| *Leptospira kirschneri* | Grippotyphosa |
| *Leptospira interrogans* | Hardjo |
| *Leptospira borgpetersenii* | Javanica |
| *Leptospira interrogans* | Kremastos |
| *Leptospira interrogans* | Medanensis |
| *Leptospira noguchii* | Panama |
| *Leptospira interrogans* | Pomona |
| *Leptospira interrogans* | Robinsoni |
| *Leptospira santarosai* | Shermani |
| *Leptospira interrogans* | Szwajizak |
| *Leptospira borgpetersenii* | Tarassovi |
| *Leptospira weilii* | Topaz |
| *Leptospira interrogans* | Zanoni |
