# Supplementary material for: Leptospirosis is an emerging infectious disease of pig-hunting dogs and humans in North Queensland
Source: PLoS Negl Trop Dis. 2022 Jan 18;16(1):e0010100. doi: 10.1371/journal.pntd.0010100 (PMC8797170; doi:10.1371/journal.pntd.0010100)
Supplement: S2 Table — (DOCX) [file pntd.0010100.s002.docx]

**S2 Table: Signalment and detailed *Leptospira* serological results of 11 pig-hunting dogs vaccinated against *Leptospira***

| Breed or type | Age (years) | Sex | *Leptospira* serovar vaccinated against | Time since last vaccination (weeks) | *Leptospira* serovars detected (>1/50) |
| --- | --- | --- | --- | --- | --- |
| Bull Arab X | 9 | M | Australis & Copenhageni | 2 | Australis (1/50);  Zanoni (1/100) |
| Bull Arab X | 6 | MN | Australis & Copenhageni | 2 | Copenhageni (1/50); Australis (1/100) |
| Pit Bull Terrier X | 7 | MN | Australis & Copenhageni | 2 | Copenhageni (1/50); Australis (1/400) |
| Bull Arab X | 6 | MN | Australis & Copenhageni | 2 | Copenhageni (1/50);  Australis (1/50);  Zanoni (1/1600);  Robinsoni (1/400) |
| Bull Arab X | 5 | M | Australis & Copenhageni | 2 | Negative |
| Bull Arab X | 0.5 | M | Australis & Copenhageni | 3 | Negative |
| Border Collie X | 11 | FN | Australis & Copenhageni | 52 | Australis (1/100) |
| Bull Arab X | 10 | FN | Australis & Copenhageni | 52 | Australis (1/200);  Zanoni (1/100);  Javanica (1/50) |
| Staghound X | 2 | MN | Australis & Copenhageni | 86 | Negative |
| Bull Arab X | 7 | MN | Australis & Copenhageni | 88 | Zanoni (1/50) |
| Bull Arab | 5 | MN | Australis | 192 | Zanoni (1/100) |

Abbreviations: M male intact; F female intact; FN spayed female; MN male castrated
