# Supplementary material for: Leptospirosis is an emerging infectious disease of pig-hunting dogs and humans in North Queensland
Source: PLoS Negl Trop Dis. 2022 Jan 18;16(1):e0010100. doi: 10.1371/journal.pntd.0010100 (PMC8797170; doi:10.1371/journal.pntd.0010100)
Supplement: S1 Text — (DOCX) [file pntd.0010100.s003.docx]

**S1 Text: Survey completed by pig-hunting dog owners**

*Determining the health and husbandry of dogs used to hunt feral pigs in Australia*

**PARTICIPANT SURVEY**

The purpose of this survey is to determine the health and husbandry of dogs used to hunt feral pigs in Australia. You have been asked to complete this survey because you own a dog that currently or has recently participated in pig hunting activities. The survey is being conducted by researchers from The University of Sydney, led by Dr. Bronwyn Orr. Please read the attached Participant Information Statement before completing this survey, as it contains important information about the study in relation to your personal data. It should take about 15 minutes to complete the survey. Your participation in this study is voluntary and you can withdraw at any time by closing your browser window. Completion and submission of the survey will be taken as your consent to use the data you provide as described above. Thank you for participating.

1. **What is your gender?**
   1. Male
   2. Female
   3. Prefer not to say
   4. Other
2. **What age bracket do you fall into?**
   1. 18-25 years
   2. 26-30 years
   3. 31-36 years
   4. 36-42 years
   5. 43-49 years
   6. 50+ years
3. **How old were you when you first went pig hunting?**
   1. Under 15 years
   2. 15-17 years
   3. 18-25 years
   4. 26-30 years
   5. 31+ years
4. **How many dogs do you have for pig hunting?**
5. **How often do you take your dogs pig hunting?**
   1. Once a year
   2. About every 3 months
   3. Monthly
   4. Every two weeks
   5. Weekly
6. **Where did you get your pig-hunting dog(s)?** (Tick the appropriate box)

|  | **Dog 1** | **Dog 2** | **Dog 3** | **Dog 4** |
| --- | --- | --- | --- | --- |
| a. Friend |  |  |  |  |
| b. Online |  |  |  |  |
| c. Shelter/ rescue |  |  |  |  |
| d. Breeder |  |  |  |  |
| e. Other (please state) |  |  |  |  |

1. **What breeds are your dogs?** (If crossbred – please indicate the breeds you can recognise as being in the mix)

| **Dog 1** | **Dog 2** | **Dog 3** | **Dog 4** |
| --- | --- | --- | --- |
|  |  |  |  |

1. **Why did you choose this dog?** (Write a few lines)

| **Dog 1** | **Dog 2** | **Dog 3** | **Dog 4** |
| --- | --- | --- | --- |
|  |  |  |  |

1. **Are your dogs bailers or luggers?**
   1. Bailers
   2. Luggers
   3. Both
2. **Where do these dogs normally live?**
   1. In your home
   2. In the house yard
   3. In kennels
   4. Combination e.g., in kennels but let out to play under supervision
   5. Other (please specify) ________________________________________
3. **Where do you normally hunt?**
   1. Queensland
   2. New South Wales
   3. Victoria
   4. Tasmania
   5. South Australia
   6. Western Australia
   7. Northern Territory
   8. Australian Capital Territory
4. **Which of the following do you have to help during pig hunting?** (Circle all that apply)
   1. Ute
   2. Dog crate on Ute
   3. Rifle
   4. GPS collars
   5. Knife
   6. Spotlights
   7. Hoist for dressing carcass
5. **How do you normally kill pigs?**
   1. Gun
   2. Knife
6. **Have you ever lost (been unable to retrieve during a hunt) a dog or dogs whilst pig hunting?**
   1. Yes
   2. No
7. **Did you ever get this dog or dogs back?**
   1. Yes
   2. No
8. **Have you ever had a dog die during or after a pig hunt because of an injury acquired during the hunt?**
   1. Yes
   2. No
9. **If yes, what did the dog die of?**
   1. Pig rip (trauma sustained from the pig)
   2. Heat stroke
   3. Snake bite
   4. Vehicle trauma
   5. Veterinary euthanasia
   6. Accidental shooting
   7. Other (please specify)___________________________________________
10. **Over the lifespan of a dog, on average how often do they get seriously injured during hunting activities?**
    1. Never
    2. Rarely
    3. About once or twice a year
    4. Almost every time we go hunting
    5. Every time we go hunting
    6. Other (please specify)_________________________________________
11. **How often do you take your dogs to the vet after they have been injured?**
    1. Rarely – I know how to care for them myself
    2. Sometimes – Only for bad injuries
    3. Often
    4. Other (please specify)_________________________________________
12. **Do you have any of the following for your dogs during hunts?** (Circle all that apply)
    1. Protective hunting collar (e.g., leather, tough fabric etc.)
    2. Stitch up kits (e.g., staple gun, bandages, gauze etc.)
    3. Chest plate (e.g., leather, tough fabric etc.)
    4. GPS unit
    5. Reflective gear
    6. Other (please specify)_________________________________________
13. **At what age do you usually retire your dogs from hunting?**
    1. 1-3 years
    2. 4-6 years
    3. 6-8 years
    4. 9+ years
14. **What do you do with dogs that are not good hunters?**
    1. Give them away
    2. Sell them online
    3. Keep them as pets (retired)
    4. Put them down (veterinary euthanasia)
    5. Shoot them
15. **What most closely describes your dogs?**
    1. Best mates
    2. Good hunters
    3. Hunting companions
    4. Family members
    5. Other (please specify)_____________________________________________
16. **Which of the following do you regularly (monthly or every 3 months) administer to your dogs?** (Select all the options that apply)
    1. Intestinal worming (e.g., Drontal, Milbemax, Interceptor, Nexgard Spectra etc.)
    2. Flea control (e.g., Frontline, Advantage, Revolution, Actyvil etc.)
    3. Tick control (e.g., Bravecto, Nextguard, Seresto collar, Killtix collar, Advantix etc.)
    4. Heartworm control (e.g., Heartgard, Interceptor, Proheart SR12, Revolution, Advocate etc.)
17. **Have your dogs ever been vaccinated?**
    1. Yes
    2. No
18. **If yes, which vaccines do they get?**
    1. Lepto (e.g., Leptospirosis, rat disease)
    2. Core (e.g., parvo, C3, Canine Parvovirus)
    3. Kennel cough (e.g., canine cough, *Bordetella bronchiseptica*)
    4. They have not been vaccinated
    5. I am not sure
    6. Other (please specify)____________________________________________
19. **How long ago did your dogs last get vaccinated?**
    1. Less than 12 months ago
    2. 1-2 years
    3. 3-5 years
    4. 6+ years
    5. Never
20. **Do you occasionally box (slaughter) pigs for human consumption?**
    1. Yes
    2. No
21. **Have you ever been vaccinated for Q Fever?**
    1. Yes
    2. No
    3. Not sure
22. **Have you ever become sick and been diagnosed with Q Fever, Brucellosis or Leptospirosis?**
    1. Yes
    2. No
    3. Not sure
23. **If yes, which disease(s) have you been diagnosed with?** (Select all the options that apply)
    1. Q Fever
    2. Brucellosis
    3. Leptospirosis
24. **Did you know that feral pigs can carry all three of these diseases?**
    1. Yes
    2. No
25. **Did you know that pig hunting dogs can become infected with all three of these diseases?**
    1. Yes
    2. No
26. **Do you take any precautions when killing pigs to avoid contracting these diseases?**
    1. Wear protective clothing (e.g., full length clothing such as jeans and a long shirt, boots etc.)
    2. Wear gloves when handling the carcass
    3. Wash hands and equipment thoroughly before eating after handling pigs
    4. Avoid internal organs of the pig (e.g., only taking the legs for meat)
    5. Other (please specify)____________________________________________________
